# Supplementary material for: Dietary Olive Leaf Extract Differentially Modulates Antioxidant Defense of Normal and Aeromonas hydrophila-Infected Common Carp (Cyprinus carpio) via Keap1/Nrf2 Pathway Signaling: A Phytochemical and Biological Link
Source: Animals (Basel). 2023 Jul 6;13(13):2229. doi: 10.3390/ani13132229 (PMC10339993; doi:10.3390/ani13132229)
Supplement: Supplementary file 1 [file animals-13-02229-s001.zip › animals-2421902-supplementary.pdf]

Supplementary Materials

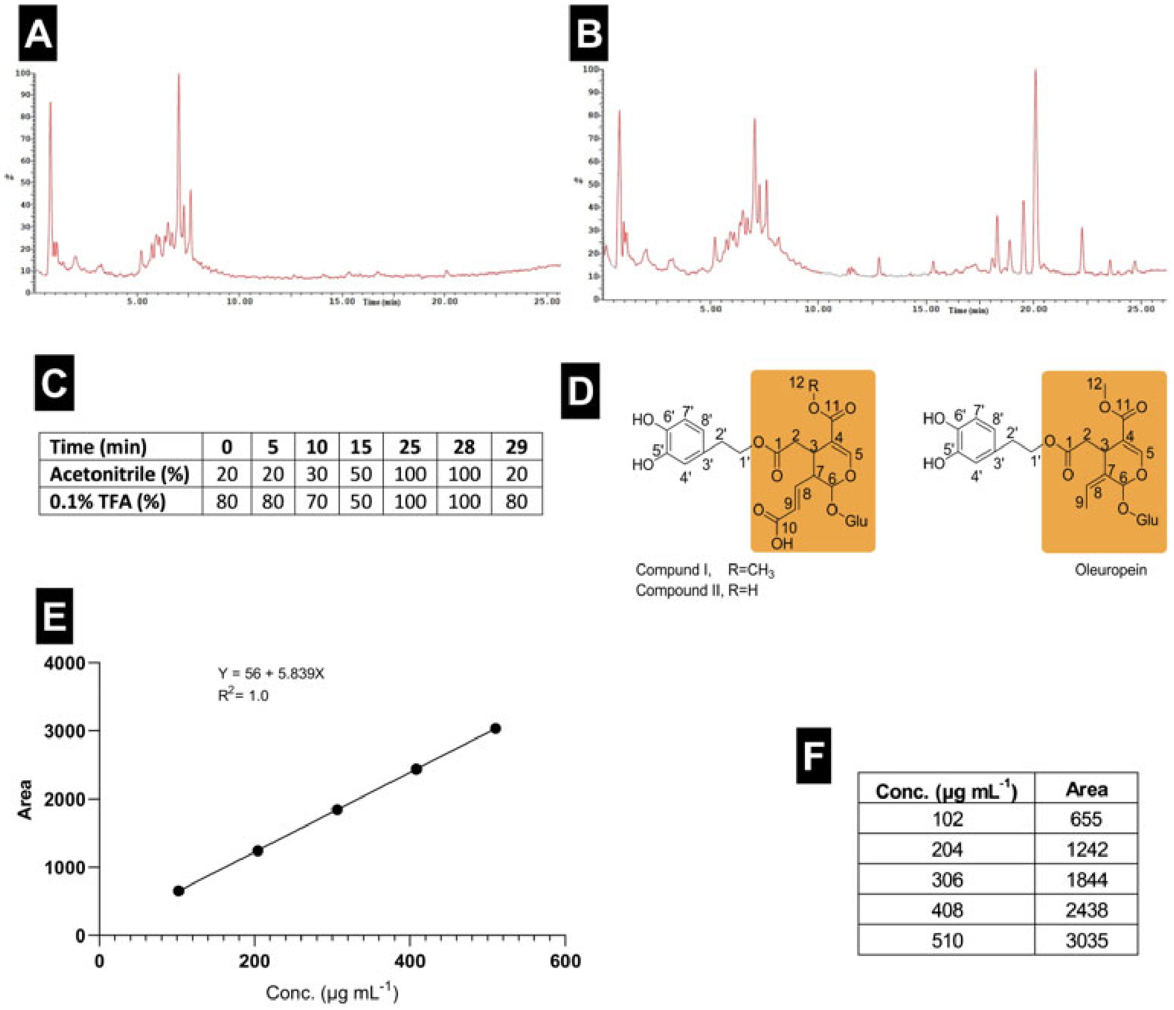

Time (min)	0	5	10	15	25	28	29
Acetonitrile (%)	20	20	30	50	100	100	20
0.1% TFA (%)	80	80	70	50	100	100	80

Conc. (µg mL^{-1})	Area
102	655
204	1242
306	1844
408	2438
510	3035

Supplementary Figure S1. HPLC analysis result of Olive leaf extract Sample of 0.546 g/100 mL was prepared Y= 5.8392X + 56. (**A**) UPLC-ESI-MS total ion chromatogram of olive leaf extract in ESI- mode. (**B**) UPLC-ESI-MS total ion chromatogram of olive leaf extract in ESI+ mode. (**C**) HPLC elution method for estimation of oleuropein. (**D**) Structures of oleuropein and isolated compounds (I and II). (**E**,**F**) Standard calibrating curve of oleuropein standard. Peak area= 6552 conc 1112.5 µg mL^{-1}, 1.1125 mg mL^{-1}, 111.25 mg/100 mL/0.546 g extract. Each 100 g contain 20.4 g **Oleuropein** (20.4 %).

Supplementary Table S1. Phytochemical profile of olive leaf extract by UPLC-ESI-MS/MS in negative and positive ion modes .

NO.	Rt min	$[M-H]^-$ m/z	$[M+H]^+$ m/z	MS^2 Ions m/z	Identification
1	0.77	195		129, 99, 75	Gluconic acid
2	0.96	191		111, 87, 85	Citric acid
3	1.25	153		141, 109	Protocatechuic
4	2.60	305		261, 243, 237, 219, 203, 191, 179,167, 165 ,139,137, 125, 111, 109, 97, 95, 81	Gallocatechin
5	3.24	593	595	447, 285, 133	Vicenin II, Luteolin-O-rutinoside
6	3.94	565		403,371, 223, 181,165,139,127,121,119 113 101, 89	Elenoic acid diglucoside
7	4.50	163		163, 117	*p*-Coumaric acid
8	4.63	609		463, 301, 300, 179, 151	Rutin
9	4.76	403		371, 359, 183, 165,151 121,119,115, 113,101	Oleoside methyl ester
10	5.20<br>5.22	 403	*427 	 371,223,181,165,139,127,121,119,113,101,89	Elenolic acid hexoside
11	5.72	525		481, 389,209,195,183,165,121,119,113,89	Demethyloleuropein
12		609		447, 285	Luteolin diglucoide
13	5.91	555		537, 403, 393, 323, 183	Hydroxy-oleuropein
14			579	271	Apigenin-7-O-rutinoside
15			595	287	Cyanidin-3-rutinoside
16	5.88	179		135, 134, 89	Caffeic acid
17		315		153, 135, 123	Hydroxytyrosol hexoside
18	6.10	623		461, 315, 179, 161	Verbascoside
19		447		301	Quercitrin
20		153		123, 109	Hydroxytyrosol
21	6.35	623		461, 315, 179, 161	Isoverbascoside
22		447		285, 175, 133	Luteolin hexoside I
23	6.35<br>22.25	543<br>284*		197, 153	Dihydro-oleuropein
24	6.50	431	433	269, 268, 117	Apigenin-7-O-glucoside
25		447		285, 284, 197, 175, 133	Luteolin hexoside II
26	6.51	701		377, 307, 175	Neo-nuzhenide
27	6.71	461	463	315, 297, 153, 135, 113	Verbasoside
28		447		285	Luteolin hexoside III
29	6.92	569		539, 407, 377	Compound II
30	7.03<br>20.11	539<br>282**		403,377,371,327,307,275,223,179,165,149,139	Oleuropein
31	7.04	359		197, 153, 135, 107	7-Deoxyloganic acid
32		137		109, 108, 93, 92	*p*-Hydroxybenzoic acid
33	7.15	539		377, 307, 275	Oleuropein isomer
34	7.26	137		107	Tyrosol
35	7.28	539		377, 307, 275	Oleuroside
36	7.61<br>11.54	523	547*<br>274**	361, 291, 259, 223, 127, 101	Ligustroside
37	8.15	925		893, 763, 745, 693, 539, 521, 377, 307	Jaspolyoside
38	8.85	375		213, 169, 151, 125, 113, 107	Loganic acid
39	9.14	377		275, 165, 149, 139, 127, 111, 95	Oleuropein aglycone
40	11.93	405		377, 275	Dimethyl oleuropein aglycone
41	18.08		425*	425	Secologanic acid
42	18.31		280**	-	Dehydro oleuropein
43	18.90		439*	439	Acetoxypinoresinol

44		455	455	Oleanoic acid
45	23.35	607	607, 541	Compound I
46	24.68	413*	413	Oleoside

* denotes for [M+Na]$^+$ adduct; ** denotes for [M+H+Na]$^+$ adduct.
